# Overcoming blame culture: key strategies to catalyse maternal and perinatal death surveillance and response

**DOI:** 10.1111/1471-0528.16989

**Published:** 2021-11-16

**Authors:** MV Kinney, LT Day, F Palestra, A Biswas, D Jackson, N Roos, A de Jonge, P Doherty, AA Manu, AC Moran, AS George, the MPDSR Technical Working Group

**Affiliations:** ^1^ School of Public Health University of the Western Cape Bellville South Africa; ^2^ Maternal Newborn Health Group Maternal Adolescent, Reproductive and Child Health (MARCH) Centre London School of Hygiene and Tropical Medicine London UK; ^3^ Maternal Newborn Health Group Department of Infectious Disease Epidemiology London School of Hygiene & Tropical Medicine London UK; ^4^ World Health Organization Geneva Switzerland; ^5^ UNFPA Dhaka Bangladesh; ^6^ Maternal, Adolescent, Reproductive and Child Health (MARCH) Centre London School of Hygiene & Tropical Medicine London UK; ^7^ Department of Medicine, Solna Clinical Epidemiology Division Karolinska Institutet Stockholm Sweden; ^8^ Karolinska University Hospital Stockholm Sweden; ^9^ Midwifery Science AVAG (Academy Midwifery Amsterdam and Groningen) Amsterdam Public Health Research Institute Amsterdam University Medical Centre Vrije Universiteit Amsterdam Amsterdam The Netherlands; ^10^ University Medical Center Groningen Groningen The Netherlands; ^11^ Options Consultancy Services Ltd St Magnus House London UK; ^12^ Epidemiology and Disease Control School of Public Health University of Ghana Legon, Accra Ghana; ^13^ Department of Maternal, Newborn, Child, Adolescent Health & Ageing World Health Organization Geneva Switzerland

## Introduction

Maternal and perinatal death surveillance and response (MPDSR) is a health systems process entailing the continuous cycle of identification, notification and review of maternal and perinatal deaths (Surveillance), followed by actions to improve service delivery and quality of care (Response).^1^ Before the coronavirus disease 2019 (COVID‐19) pandemic, there were an estimated 4.6 million maternal and neonatal deaths and stillbirths each year.^2^ During the pandemic, maternal and perinatal health outcomes have worsened, especially in low‐ and middle‐income countries,^3^ highlighting the urgent need to galvanise MPDSR to end preventable mortality and strengthen health systems.

The World Health Organization (WHO) has released global technical guidelines on MPDSR with operational guidance and tools,^4^ and has listed it among the essential interventions to mitigate the indirect effects of COVID‐19 on maternal and perinatal outcomes.^5^ As countries adapt and apply these guidance, implementation gaps and challenges remain preventing successful MPDSR uptake.^1^ The organisational climate and culture relating to MPDSR, including elements of blame, have been identified as key factors requiring further attention.^1^, ^6^, ^7^, ^8^ This commentary presents strategies to identify, address and overcome the blame culture relating to MPDSR. It builds from Lewis’s 2014 framework on the cultural environment of maternal death and near‐miss reviews published in the *BJOG* 2014 supplement on quality of care.^8^

## The importance of a blame‐free, confidential climate

MPDSR implementation is affected by factors at multiple health system levels^8^:

1
*Individual responsibility for, and ownership of, the MPDSR process (micro level)* whereby health workers embrace positive attitudes of life‐long learning for behaviour change to improve maternal and perinatal health.^8^ MPDSR implementation relies on health workers’ commitment to lead in the process and participate in peer‐discussion to identify modifiable factors, and for individuals and teams to be willing to change and implement solutions.^7^
2
*Organisational culture (meso level)* whereby the health facility’s work environment influences implementation.^8^ MPDSR succeeds when there is an organisational culture of learning as a critical part of quality improvement.^1^
3
*Policy and political supportive environment (macro level)* whereby national policies initiate and support MPDSR implementation, including guidelines, and legal and other protective frameworks. Implementation is facilitated by political priority for maternal and neonatal health with corresponding investment to deliver quality services.^1^, ^9^

Across all three levels, successful implementation of MPDSR requires a ‘No Name, No Blame and No Shame’ environment, which is grounded in three ethical principles: confidentiality, anonymity and respect. The concept of blame relating to MPDSR is complex; taking different forms, arising for different reasons and with varying perspectives between settings.^1^ ‘No blame’ is integral to ‘No name’ and ‘No shame’ in MPDSR and if a blame culture persists, MPDSR efforts will fail.

‘Blame culture’ linked to MPDSR widely exists at the micro and meso levels.^1^ Individuals can feel threatened during MPDSR review meetings – fearing punitive repercussions and legal action.^1^ Health‐worker emotional fatigue and burnout with high workloads, exacerbated by the pandemic, can further exacerbate the culture of blame. The negative influence of professional hierarchies between health cadres can silence nurse‐midwives and junior medical staff,^6^ and may even demotivate personnel from participating in MPDSR. Other contributing factors include a lack of clarity around the ‘no name, no blame, no shame’ principle, defensiveness regarding poor quality record‐keeping, poor facilitation of review meetings and lack of staff time to participate.^1^ Ineffective management, communication and coordination across teams may also constrain the MPDSR process, when management or senior team members do not buy into or engage in the process. Finally, without national political commitment, government and clinical setting ownership and clear guidelines, MPDSR implementation will face many challenges.^9^

## A framework for promoting a positive implementation culture of MPDSR

**Figure 1:**
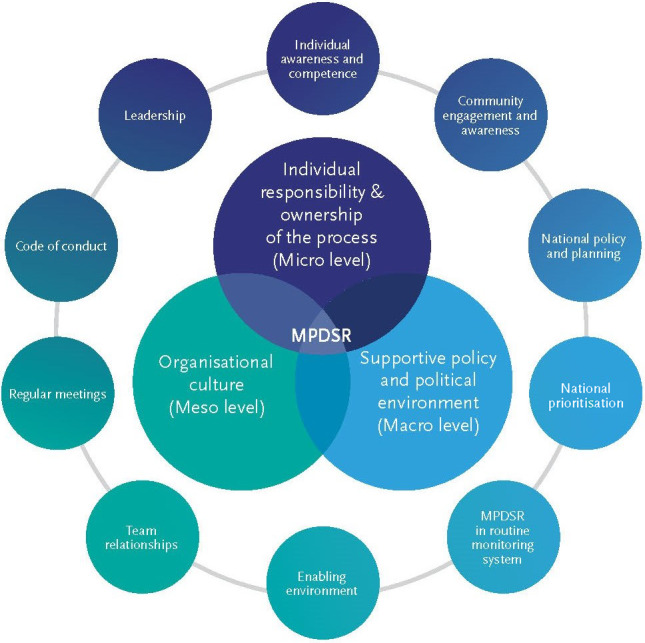
Framework for overcoming blame culture to promote a positive implementation culture for MPDSR. *Source*: WHO Maternal and Perinatal Death Surveillance and Response: Materials to Support Implementation. Working document August 2021.^10^

Despite the identification of some strategies to overcome the blame culture previously,^8^ blame remains a major barrier to effective implementation.^1^ To support frontline health workers, managers and planners at all levels to overcome this challenge, we present ten strategies using an adapted framework to promote a positive implementation culture of MPDSR (Figure 1). Adapted from Lewis,^8^ further investigated^1^ and vetted by the MPDSR Global Technical Working Group, the ten strategies integrate micro, meso and macro levels of the health system to reduce blame culture. This framework has also been included in the new WHO materials to support MPDSR implementation.^10^

## Strategies to minimise the blame culture

This section explains the ten strategies, with further information in Table S1. Boxes 1, 2, 3 provide country case studies of these strategies in practice, revealing how they are also interlinked.^11^, ^12^

1
*Ensure MPDSR policy and planning* including national guidelines that clearly explain the purpose, process and how to conduct blame‐free MPDSR with implementation tools available at all levels of the health system. Policies for death notification requirements and legal protection for individual staff and health departments linked to MPDSR need careful consideration. Fear of litigation has been reported as potentially helpful for positive accountability, as well as a negative influence.^1^
2
*Ensure national prioritisation* of ending preventable maternal and neonatal deaths and stillbirths, leading to positive promotion and use of MPDSR. Prioritisation is especially critical during the COVID‐19 pandemic to assure that team’s monitor and mitigate potential health system challenges. Although political commitment can result in increased MPDSR implementation,^6^ it may also lead to additional pressure on over‐burdened health workers compromising MPDSR accurate reporting and participation.^1^ Therefore, dual national prioritisation on the value of systems learning and quality improvement that MPDSR encompasses needs to be matched with political priority for health system investment to implement *response*, deliver improved health outcomes and reduce the number of preventable deaths.
3
*Harmonise MPDSR with routine monitoring systems* to support process standardisation and strengthen accountability. Integrating elements of MPDSR within routine monitoring systems, e.g. data collection, aims to increase efficiency and sustainability by reducing duplicative data capture and workload. Enabling real‐time regular data use may ultimately result in less blame as MPDSR becomes normalised as part of routine data systems, and can serve as a means of verifying data across systems.
4
*Create and advocate for an enabling environment* that supports MPDSR implementation with an organisational culture of learning, accountability and transparency. Enabling environment means that health system building blocks are functioning, i.e. adequate human and physical resources, along with other elements, such as coordinating mechanisms, supportive relationships and quality improvement strategies. During the pandemic, advocacy for the continued need for MPDSR systems to operate with adequate resourcing and staffing is essential to allow health systems to respond to their current context, including identification of service delivery disruption and worsening of COVID‐19 in specific geographical areas. Advocating for an enabling environment that supports continued implementation during pandemics and in routine contexts protects staff from burn out and blame. Specific to the review process, promoting a learning focus and anonymity mitigates blame.^1^ Reviewing cases of newborn survival and near‐miss maternal deaths can change the review meeting’s atmosphere to further alleviate blame tendencies while celebrating team success. Provision of incentives, such as refreshments and continuous capacity building, may strengthen overall implementation efforts.
5
*Strengthen leadership* within professional cadres participating in MPDSR at all levels. A culture of trust is nurtured by strong inter‐professional leadership and continuous modelling of a ‘blame‐free culture’.^6^ It is critical that MPDSR focal persons have high technical competence, and that the chairperson of the review meeting is an experienced facilitator to model blame‐free and educational approaches. MPDSR champions or engaged leaders are often highly motivated senior staff who already serve as mentors and in supportive supervisory roles.
6
*Nurture team relationships* among MPDSR participants. Teams with healthy relationships take collective responsibility and support one another. A teamwork approach to MPDSR facilitates consensus around decision‐making, inclusiveness, strong supportive supervision and delegation of responsibility to implement solutions.^1^ Health facility management plays a strong role in strengthening team relationships for MPDSR through clear communication and their involvement and support in MPDSR. Specific attention is needed to strengthen team relationships during the pandemic as the additional strain on the health workforce can lead to emotional exhaustion and possibly a lack of empathy of healthcare workers towards mothers and each other, which could contribute to the blame culture.
7
*Ensure that regular multidisciplinary review meetings take place* to embed MPDSR in routine practice. Continuous engagement and frequent positive experiences of MPDSR review meetings can reinforce the ‘no blame’ culture (Box 1, Panel S1). Participation of all health‐worker cadres caring for women and newborns, including junior and senior team members, creates ownership, enhances the discussion, strengthens the response and reinforces non‐blame teamwork. Active participation of all cadres can reinforce the centrality of inter‐professional teamwork across hierarchies.^10^
8
*Establish a code of conduct or ‘audit charter’* for review meetings to ensure clear understanding about the purpose of the meeting, expected behaviour (‘no name, no blame, no shame’) and confidentiality. Codes of conduct may minimise acrimony and prevent (or reduce) blame.^1^ In some settings, a code of conduct would be a signed or verbally agreed non‐disclosure confidentiality agreement (Box 2, Panel S2).
9
Promote *individual awareness* of everyone’s role, responsibility and competence to ensure a ‘No Name, No Blame and No Shame’ process. Every participant engaged in MPDSR needs to understand the MPDSR purpose and process, and to take ownership and responsibility for jointly implementing solutions identified to avert future deaths. Individual awareness can be improved through ongoing engagement in the process as on‐the‐job capacity development.
10
*Engage communities in awareness* reporting and participation in MPDSR cycles, where appropriate. Community awareness and engagement may strengthen collective ownership and responsibility, and ultimately improve quality of care.^9^ Regular feedback of results to communities may also ensure accountability and promotes sustainability.^1^, ^6^ Building community awareness and sensitisation around the MPDSR process, for example through social autopsies, may create an enabling environment for implementation at community level. Critical here will be emphasising the ‘No Name, No Blame and No Shame’ approach so that family, community members and health workers are able to discuss openly and constructively how similar deaths can be prevented in the future (Box 3, Panel S3).

Box 1 **Multidisciplinary participation in Zimbabwe**

**Table:** 

Multidisciplinary participation can reduce blame because more people are engaged in the discussion and can share their perspectives. An assessment of MPDSR implementation in 16 facilities across Zimbabwe found evidence of multidisciplinary participation in death audit meetings with clinical staff from different units (obstetrics, paediatrics, unit in charge) as well as hospital administration, such as information officers, hospital and district management and community liaisons. The interdisciplinary nature of audit meetings demonstrated buy‐in and ownership in the process by all staff and reflected strong facility leadership. The assessment also found that there was little fear or blame associated with death review meetings reported. Only six facilities reported a connection to professional disciplinary action and the MPDSR system. In order to ensure separation between these systems, adopting a mortality audit meeting code of conduct that clearly differentiates between mortality audit and professional disciplinary or legal processes can help to give staff greater confidence to share openly with less fear of punishment or blame, as displayed in the below quotes.
*‘Everyone attends our maternal and perinatal meetings, all the way to the driver, because when we have a case to transfer, he knows why we need to move now.’ – Facility interview, Zimbabwe*.
*We make sure we don’t say the names of those who attended the patient. No one says, “I am the one.” Just “doctor” or “nurse.” — Facility interview, Zimbabwe*.

Source: Kinney et al.^11^

Box 2 **Code of conduct and staff protection in Tanzania**

**Table:** 

The National MPDSR guideline in Tanzania stipulates that a facility should have a code of conduct for MPDSR. In an assessment of MPDSR implementation across 16 facilities in Tanzania, respondents reported that they adhere to the code of conduct. However, the document review and interviews found inconsistency and poor documentation of an actual code of conduct in all but three facilities. Two of these facilities reported that the MPDSR meeting chairperson reads the code before starting the meeting, which was validated through document review. At the third facility, the code of conduct was embedded in the letter to staff inviting them to join the MPDSR committee members (see extract from letter below). These three facilities demonstrated leadership by hospital management to promote an organisational culture of participation. Although the other facilities in the assessment could not show the use of codes of conduct in their meetings, three‐quarters of health facilities had measures to ensure staff confidentiality and did not include names in the review notes.
Extract from the letter inviting staff to join the MPDSR committee: *‘The main objective of the committee is to discuss all maternal and perinatal death, which will happen to occur in our hospital and to make action plan for better improvement of maternal and perinatal care at our hospital as well as at the district level. This team will seat for discussion within seven days after occurrence of maternal or perinatal death*.
*The rule of the Team is* *To arrive on time for the review session*. *To respect the statements and ideas of everyone*. *To respect the confidentiality of the team discussions and information and problems raised during the review must not be communicated outside the team*. *To participate actively in the discussion*. *To accept discussion and debate among participants without verbal violence*. *To refrain from hiding or falsifying information that could be useful in understanding the case being reviewed*. *To accept that our own action/decision may be questioned.”*
*(Health facility document review, Tanzania, data collected in May 2017)*

Source: Kinney et al.^11^

Box 3 **The importance of community engagement to reduce blame**

**Table:** 

In settings where many births occur outside the health facility, it is difficult to get accurate reporting of maternal and perinatal deaths. Issues around fear of blame often prevent reporting of deaths by family members, health workers or traditional birth attendants who were involved in treating the woman or newborn. Community engagement in MPDSR, when facilitated well, can help minimise blame by involving various members of the community and emphasising the need to address systemic issues rather than individual fault.
The Government of Bangladesh introduced social autopsy in 2010 to engage the communities in examining the social determinants of a maternal death, neonatal death or stillbirth through a guided, structured, standardised analysis. After a decade of implementation, social autopsy has enabled stronger data collection of social causes behind deaths, as well as empowered communities to identify their own problems, identify solutions and take appropriate action. Ensuring a blame‐free environment has led to successful implementation through open discussions about cases. In order to foster a blame‐free environment, the following steps have been taken in Bangladesh when implementing social autopsy:
The facilitator of the meeting receives adequate training on social autopsy, including facilitation skills to avoid blame in the meeting. The facilitator is someone who is familiar to the community, ideally someone who works in the area where the death occurred, which allows participants to feel confident and comfortable discussing these issues in front of government health workers. Prior to the social autopsy session, the bereaved family and other participants are briefed on the process, and consent is requested. Before starting the session, the facilitator describes the objectives and expected outcome of the social autopsy. Throughout the session, the facilitator steers the discussion to avoid any blame on any person, provider or institution.

Source: Mahato et al.^12^

## Conclusion

The COVID‐19 pandemic highlights the urgent need to further strengthen MPDSR as part of the effort to reach the Sustainable Development Goals to end preventable maternal and neonatal deaths and stillbirths and improve health service delivery. Overcoming the blame culture that currently impedes MPDSR implementation requires action at all levels of the health system. Targeted strategies across the health system will create a healthier culture and environment for implementing MPDSR. Future research needs to go beyond identifying blame as a barrier, to understanding how effectively these strategies can change the blame culture across diverse contexts to scale‐up MPDSR, strengthen health systems and ultimately save lives and prevent suffering.

### Disclosure of interests

None declared. Completed disclosure of interests form available to view online as supporting information.

### Contribution of authorship

This commentary was prepared by the MPDSR Technical Working Group’s subgroup assigned to further understand the blame culture. MVK, LTD, FP, DJ and AM conceptualised the idea. MVK wrote the first draft with inputs from LTD and DJ. All authors reviewed and provided edits to the manuscript. FP, AM and ASG supervised the process. All authors reviewed and approved the final version.

### Details of ethics approval

This work is a commentary based on the literature and is not a scientific study in itself. Institutional Review Board approval was not required from any of the authors’ institutions.

### Funding

Asha George and Mary Kinney are supported by the South African Research Chair's Initiative of the Department of Science and Technology and National Research Foundation of South Africa (Grant No. 82769), the South African Medical Research Council and the Countdown 2030 project funded by the Bill and Melinda Gates Foundation. Any opinion, finding and conclusion or recommendation expressed in this material is that of the authors and funders do not accept any liability in this regard. The authors alone are responsible for the views expressed in this article and they do not necessarily represent the views, decisions or policies of the institutions with which they are affiliated.

## Supporting information


**Table S1**. Ten strategies for promoting a ‘No Name, No Blame and No Shame’ culture and key resources with more information.
**Panel S1**. Example of principles of facility‐based case review meetings to ensure no blame.
**Panel S2**. Examples of audit charter or non‐disclosure agreements.
**Panel S3**. Engaging the community to prevent blame.

Supplementary Material

Supplementary Material

Supplementary Material

Supplementary Material

Supplementary Material

Supplementary Material

Supplementary Material

Supplementary Material

Supplementary Material

Supplementary Material

## Data Availability

Data sharing not applicable to this article as no data sets were generated or analysed during the current study.
