# Supplementary material for: Overcoming blame culture: key strategies to catalyse maternal and perinatal death surveillance and response
Source: BJOG. 2021 Nov 16;129(6):839–44. doi: 10.1111/1471-0528.16989 (PMC9298870; doi:10.1111/1471-0528.16989)
Supplement: Supplementary file 1 — Table S1. Ten strategies for promoting a ‘No Name, No Blame and No Shame’ culture and key resources with more information. Panel S1. Example of principles of facility‐based case review meetings to ensure no blame. Panel S2. Examples of audit charter or non‐disclosure agreements. Panel S3. Engaging the community to prevent blame. [file BJO-129-839-s008.docx]

Supporting Information

**Title**: Overcoming blame culture: Key strategies to catalyze Maternal and Perinatal Death Surveillance and Response

Shortened running title (<60 characters): Overcoming blame culture of MPDSR

**Authors**: Kinney MV, Day LT, Palestra F, Biswas A, Jackson D, Roos N, de Jonge A, Doherty P, Manu A, Moran, AC, George AS, on behalf of the MPDSR Technical Working Group

Contents:

[Table S1. Ten strategies for promoting a “No Name, No Blame and No Shame” culture and key resources with more information 1](#_Toc79744568)

[Panel S1. Example of principles of facility-based case review meetings to ensure no blame 10](#_Toc79744569)

[Panel S2. Examples of audit charter or non-disclosure agreements 11](#_Toc79744570)

[Panel S3: Engaging the community to prevent blame 11](#_Toc79744571)

## Table S1. Ten strategies for promoting a “No Name, No Blame and No Shame” culture and key resources with more information

| **Strategy** | **Markers or measures** | **Level** | **Key literature** |
| --- | --- | --- | --- |
| **Ensure that policy and planning for MPDSR includes national guidelines and policies** on how to conduct blame-free MPDSR, and legal frameworks to draw a distinction between the audit process and appropriate disciplinary action | Policy mandate: national MPDSR policy and guidelines  Availability of MPDSR tools  Legal framework for notifying deaths and involve communities and other sectors | Macro | Smith H, Ameh C, Godia P, Maua J, Bartilol K, Amoth P et al. Implementing maternal death surveillance and response in Kenya: incremental progress and lessons learned. Glob Health Sci Pract. 2017;5(3):345–54.  WHO Global Reproductive, Maternal, Newborn, Child and Adolescent Health Policy Survey: indicates which countries have national MPDSR guidelines (<https://www.who.int/data/maternal-newborn-child-adolescent-ageing/national-policies?selectedTabName=Documents>, accessed 29 May 2021).  E4A. 2012. Maternal death surveillance and response systems: overcoming legal challenges and creating an enabling environment. MDSR Action Network. Presented during ‘Interactive MDSR Resource Room’ at XXFIGO World Congress of Gynecology and Obstetrics, Rome, Italy, 7-12 October 2012. |
| Ensure **national prioritization** of prevention of maternal and perinatal deaths | Global and regional commitments (e.g. SDGs)  Targets in national health plans | Macro | Melberg A, Mirkuzie AH, Sisay TA, Sisay MM, Moland KM. "Maternal deaths should simply be 0": politicization of maternal death reporting and review processes in Ethiopia. Health Policy Plan. 2019;34(7):492–8.  Tura AK, Fage SG, Ibrahim AM, Mohamed A, Ahmed R, Gure T et al. Beyond No Blame: practical challenges of conducting maternal and perinatal death reviews in eastern Ethiopia. Glob Health Sci Pract. 2020;8(2):150–4. doi:10.9745/GHSP-D-19-00366. |
| **Harmonize MPDSR in** **routine monitoring systems** to standardize the process and enable accountability | Integrating MPDSR into DHIS or other national routine monitoring systems | Macro, meso, micro | Biswas A. Shifting paradigm of maternal and perinatal death review system in Bangladesh: a real-time approach to address sustainable developmental goal 3 by 2030. F1000Res. 2017;6:1120.  Purandare C, Bhardwaj A, Malhotra M, Bhushan H, Shah PK. Every death counts: electronic tracking systems for maternal death review in India. Int J Gynaecol Obstet. 2014;127(Suppl 1):S35–9.  Smith H, Ameh C, Godia P, Maua J, Bartilol K, Amoth P et al. Implementing maternal death surveillance and response in Kenya: incremental progress and lessons learned. Glob Health Sci Pract. 2017;5(3):345–54. |
| Create and advocate for an **overall enabling environment for implementation**, including an organizational culture of learning, accountability and transparency | Address human and material resource shortages across the system  Coordination mechanisms  Implementing broader quality improvement strategies  Data qquality assessments  Promote MPDSR as a learning experience  Prioritize preventative measures  Ensure anonymity – e.g. notes and reports – to protect patients and staff involved | Macro, meso | Austin A, Langer A, Salam RA, Lassi ZS, Das JK, Bhutta ZA. Approaches to improve the quality of maternal and newborn health care: an overview of the evidence. Reprod Health. 2014;11(Suppl 2):S1. doi: 10.1186/1742-4755-11-S2-S1.  Bandali, S., Thomas, C., Hukin, E., Matthews, Z., Mathai, M., Ramachandran Dilip, T., Roos, N., Lawley, R., Igado, O. & Hulton, L. 2016. Maternal Death Surveillance and Response Systems in driving accountability and influencing change. Int J Gynaecol Obstet, 135, 365-371.  Belizan M, Bergh AM, Cilliers C, Pattinson RC, Voce A & for the Synergy Group. Stages of change: a qualitative study on the implementation of a perinatal audit programme in South Africa. BMC Health Serv Re*s.* 2011;11:243.  Bergh AM, Pattinson R, Belizan M, Cilliers C, Jackson D, Kerber K et al. & for the Synergy Group. Completing the audit cycle for quality care in perinatal, newborn and child health. Pretoria: Medical Research Council of South Africa; 2011.  Biswas, A., Rahman, F., Eriksson, C., Halim, A. & Dalal, K. 2015. Facility Death Review of Maternal and Neonatal Deaths in Bangladesh. PLoS One, 10, e0141902.  de Kok B, Imamura M, Kanguru L, Owolabi O, Okonofua F, Hussein J. Achieving accountability through maternal death reviews in Nigeria: a process analysis. Health Policy Plan. 2017;32(8):1083–91.  Dumont A, Tourigny C, Fournier P. Improving obstetric care in low-resource settings: implementation of facility-based maternal death reviews in five pilot hospitals in Senegal. Hum Resour Health. 2009;7: 61.  Lewis G. The cultural environment behind successful maternal death and morbidity reviews. BJOG. 2014;121(Suppl 4):24-31.  Lewis G. Emerging lessons from the FIGO LOGIC initiative on maternal death and near-miss reviews. Int J Gynaecol Obstet. 2014;127(Suppl 1):S17–20.  Manandhar, D. S. 2004. Perinatal death audit. Kathmandu Univ Med J (KUMJ), 2, 375-83.  Richard F, Ouedraogo C, Zongo V, Ouattara F, Zongo S, Gruénais ME et al. The difficulty of questioning clinical practice: experience of facility-based case reviews in Ouagadougou, Burkina Faso. BJOG. 2009;116(1):38–44. |
| **Strengthen leadership** within all participating professional groups at all levels, ensuring engagement with the MPDSR focal point on how to facilitate meetings and mentor others | Facilitation skills for conducting audit meetings  Mentorship and supportive supervision  Participation of senior staff in the meetings and in the data analysis in order to guide priorities and actions | Meso, Micro | Bakker, W., van den Akker, T., Mwagomba, B., Khukulu, R., van Elteren, M. & van Roosmalen, J. 2011. Health workers' perceptions of obstetric critical incident audit in Thyolo District, Malawi. Trop Med Int Health, 16, 1243-50.  Bergh AM, Pattinson R, Belizan M, Cilliers C, Jackson D, Kerber K et al. & for the Synergy Group. Completing the audit cycle for quality care in perinatal, newborn and child health. Pretoria: Medical Research Council of South Africa; 2011.  Dumont A, Tourigny C, Fournier P. Improving obstetric care in low-resource settings: implementation of facility-based maternal death reviews in five pilot hospitals in Senegal. Hum Resour Health. 2009;7:61.  Kinney MV, Ajayi G, de Graft-Johnson J, Hill K, Khadka N, Om’Iniabohs A et al. "It might be a statistic to me, but every death matters": an assessment of facility-level maternal and perinatal death surveillance and response systems in four sub-Saharan African countries. PloS One. 2020;15(12):e0243722.  Koblinsky M. Maternal death surveillance and response: a tall order for effectiveness in resource-poor settings. Glob Health Sci Pract. 2017;5:333–7.  MCSP. Assessment of Maternal and Perinatal Death Surveillance and Response Implementation in Nigeria. Washington (DC): Maternal Child Survival Program; 2017.  MCSP. Assessment of Maternal and Perinatal Death Surveillance and Response Implementation in Rwanda. Washington (DC): Maternal Child Survival Program; 2017.  MCSP. Assessment of Maternal and Perinatal Death Surveillance and Response Implementation in Zimbabwe. Washington (DC): Maternal Child Survival Program; 2017.  MCSP. Assessment of Maternal and Perinatal Death Surveillance and Response Implementation in Kagera and Mara Region, Tanzania. Washington (DC): Maternal Child Survival Program; 2017.  Purandare C, Bhardwaj A, Malhotra M, Bhushan H, Shah PK. Every death counts: electronic tracking systems for maternal death review in India. Int J Gynaecol Obstet. 2014;127(Suppl 1):S35–9.  Rhoda NR, Greenfield D, Muller M, et al. Experiences with perinatal death reviews in South Africa – the Perinatal Problem Identification Programme: scaling up from programme to province to country. BJOG. 2014;121(Suppl 4):160–6.  van Hamersveld KT, den Bakker E, Nyamtema AS, van den Akker T, Mfinanga EH, van Elteren M et al. Barriers to conducting effective obstetric audit in Ifakara: a qualitative assessment in an under-resourced setting in Tanzania. Trop Med Int Health. 2012;17(5):652–7. |
| **Nurture team relationships** among those who participate in audit through continuous engagement, a teamwork approach, support from hospital management, deliberate efforts and strategies, such as mentorship | Mentorship, clinical outreach and supervisory activities through district engagement  Teams: committees formed and multidisciplinary  Relationship between committee members | Meso, Micro | Agaro C, Beyeza-Kashesya J, Waiswa P, Sekandi JN, Tusiime S, Anguzu R et al. The conduct of maternal and perinatal death reviews in Oyam District, Uganda: a descriptive cross-sectional study. BMC Womens Health. 2016;16:38.  Dartey AF. The role of midwives in the implementation of maternal death review (MDR) in health facilities in Ashanti region, Ghana. Cape Town: University of the Western Cape; 2012.  Hofman JJ, Mohammed H. Experiences with facility-based maternal death reviews in northern Nigeria. Int J Gynaecol Obstet. 2014;126:111–4.  MCSP 2017. Assessment of Maternal and Perinatal Death Surveillance and Response Implementation in Nigeria. Washington, DC: Maternal Child Survival Program.  MCSP 2017. Assessment of Maternal and Perinatal Death Surveillance and Response Implementation in Rwanda. Washington, DC: Maternal Child Survival Program.  MCSP 2017. Assessment of Maternal and Perinatal Death Surveillance and Response Implementation in Zimbabwe. Washington, DC: Maternal Child Survival Program.  MCSP 2018. Assessment of Maternal and Perinatal Death Surveillance and Response (MPDSR) Implementation in Kagera and Mara Region, Tanzania. Washington, DC: Maternal Child Survival Program.  Muffler N, Trabelssi Mel H, De Brouwere V. Scaling up clinical audits of obstetric cases in Morocco. Trop Med Int Health. 2007;12(10):1248–57.  Purandare C, Bhardwaj A, Malhotra M, Bhushan H, Shah PK. Every death counts: electronic tracking systems for maternal death review in India. Int J Gynaecol Obstet. 2014;127(Suppl 1):S35–9. |
| **Ensure that audit meetings take place regularly** and staff regularly attend. The literature shows that the more people attend, the more practice they have and the more embedded the process becomes | Meeting frequency  Incentivize attendance – e.g. staff receive professional credit points for participation or attending meetings is part of work expectations | Meso | Dartey AF. The role of midwives in the implementation of maternal death review (MDR) in health facilities in Ashanti region, Ghana. Cape Town: University of the Western Cape; 2012.  Kinney MV, Ajayi G, de Graft-Johnson J, Hill K, Khadka N, Om’Iniabohs A et al. "It might be a statistic to me, but every death matters": an assessment of facility-level maternal and perinatal death surveillance and response systems in four sub-Saharan African countries. PloS One. 2020;15(12):e0243722.  Lewis G. Emerging lessons from the FIGO LOGIC initiative on maternal death and near-miss reviews. Int J Gynaecol Obstet. 2014;127(Suppl 1):S17–20.  MCSP 2017. Assessment of Maternal and Perinatal Death Surveillance and Response Implementation in Zimbabwe. Washington, DC: Maternal Child Survival Program.  Tayebwa E, Sayinzoga F, Umunyana J, et al. Assessing Implementation of Maternal and Perinatal Death Surveillance and Response in Rwanda. International journal of environmental research and public health 2020; 17(12). |
| Put in place a **code of conduct or “audit charter”** with clear rules about the purpose of meetings, expected behaviour during meetings and the confidentiality of meetings | Staff involved in MPDSR commit to never sharing the information  Review committee members sign or verbally consent to a non-disclosure confidentiality agreement  Publication of proceedings are anonymous | Meso | Congo B, Sanon D, Millogo T, Ouedraogo CM, Yaméogo WME, Meda ZC et al. Inadequate programming, insufficient communication and non-compliance with the basic principles of maternal death audits in health districts in Burkina Faso: a qualitative study. Reprod Health. 2017;14(1):121.  Kinney MV, Ajayi G, de Graft-Johnson J, Hill K, Khadka N, Om’Iniabohs A et al. "It might be a statistic to me, but every death matters": an assessment of facility-level maternal and perinatal death surveillance and response systems in four sub-Saharan African countries. PloS One. 2020;15(12):e0243722.  Lewis G. Emerging lessons from the FIGO LOGIC initiative on maternal death and near-miss reviews. Int J Gynaecol Obstet. 2014;127(Suppl 1):S17–20.  Richard F, Ouedraogo C, Zongo V, Ouattara F, Zongo S, Gruénais ME et al. The difficulty of questioning clinical practice: experience of facility-based case reviews in Ouagadougou, Burkina Faso. BJOG. 2009;116(1):38–44. |
| Promote **individual awareness** of roles and responsibilities, and **competence** to complete tasks through on-the-job capacity-development linked to implementation of a non-blaming approach | Competencies of managers, supervisors, providers to analysis and interpret data and information  Confidence of and capability of health workers to complete and analyse deaths  Strategy for staff orientation to MPDSR | Micro | Armstrong CE, Lange IL, Magoma M, Ferla C, Filippi V, Ronsmans C. Strengths and weaknesses in the implementation of maternal and perinatal death reviews in Tanzania: perceptions, processes and practice. Trop Med Int Health. 2014;19:1087–95.  Belizan M, Bergh AM, Cilliers C, Pattinson RC, Voce A & for the Synergy Group. Stages of change: a qualitative study on the implementation of a perinatal audit programme in South Africa. BMC Health Serv Re*s.* 2011;11:243.  Bergh AM, Pattinson R, Belizan M, Cilliers C, Jackson D, Kerber K et al. & for the Synergy Group. Completing the audit cycle for quality care in perinatal, newborn and child health. Pretoria: Medical Research Council of South Africa; 2011.  Muffler, N., Trabelssi Mel, H. & De Brouwere, V. 2007. Scaling up clinical audits of obstetric cases in Morocco. Trop Med Int Health, 12, 1248-57.  Richard F, Ouedraogo C, Zongo V, Ouattara F, Zongo S, Gruénais ME et al. The difficulty of questioning clinical practice: experience of facility-based case reviews in Ouagadougou, Burkina Faso. BJOG. 2009;116(1):38–44. |
| **Engage communities** **in awareness** about reporting and participation in MPDSR verbal and social autopsies | Building community awareness and community sensitization.  Create an enabling environment for community MPDSR  Informed consent to ensure freedom of community to speak and ethics  Confidentiality,  engagement and relationship between health-care providers and the community.  Prioritize role of MPDSR focal person to facilitate community MPDSR  Social autopsy serves as a health promotion tool for the community to address maternal and perinatal death | Micro | Bayley O, Chapota H, Kainja E, Phiri T, Gondwe C, King C, Nambiar B, Mwansambo C, Kazembe P, Costello A, Rosato M, Colbourn T. Community-linked maternal death review (CLMDR) to measure and prevent maternal mortality: a pilot study in rural Malawi. BMJ Open. 2015 Apr 20;5(4):e007753. doi: 10.1136/bmjopen-2015-007753. PMID: 25897028; PMCID: PMC4410129.  Biswas A, Ferdoush J, Abdullah ASM, Halim A. Social autopsy for maternal and perinatal deaths in Bangladesh: a tool for community dialog and decision making. Public Health Rev. 2018;39(1).  Biswas A. Shifting paradigm of maternal and perinatal death review system in Bangladesh: a real-time approach to address sustainable developmental goal 3 by 2030. F1000Res. 2017;6:1120.  Biswas A, Halim MA, Dalal K, Rahman F. Exploration of social factors associated to maternal deaths due to haemorrhage and convulsions: analysis of 28 social autopsies in rural Bangladesh. BMC Health Serv Res. 2016;16(1):659.  Biswas A, Rahman F, Eriksson C, Halim A, Dalal K. Social autopsy of maternal, neonatal deaths and stillbirths in rural Bangladesh: qualitative exploration of its effect and community acceptance. BMJ Open. 2016;6(8):e010490.  Biswas A, Rahman F, Halim A, Eriksson C, Dalal K. Experiences of community verbal autopsy in maternal and newborn health of Bangladesh. HealthMED. 2015;9(8):329–38.  Biswas A, Rahman F, Eriksson C, Halim A, Dalal K. Facility death review of maternal and neonatal deaths in Bangladesh. PLoS One. 2015;10(11):e0141902.  Biswas A, Rahman F, Eriksson C, Dalal K. Community notification of maternal, neonatal deaths and still births in Maternal and Neonatal Death Review (MNDR) system: experiences in Bangladesh. Health. 2014;6(6):2218–26.  Biswas A, Rahman F, Halim A, Eriksson C, Dalal K. Maternal and Neonatal Death Review (MNDR): a useful approach to Identifying appropriate and effective maternal and neonatal health initiatives in Bangladesh. 2014;6:1669–79.  Biswas A. Social autopsy as an intervention tool in the community to prevent maternal and neonatal deaths: experiences from Bangladesh. MDSR Action Network. June 2016.  Biswas A. Maternal and Perinatal Death Review (MPDR): experiences in Bangladesh. Geneva: World Health Organization; 2015 (<http://www.who.int/maternal_child_adolescent/epidemiology/maternal-death-surveillance/case-studies/bangladesh-study/en/>, accessed 29 May 2021).  Halim A, Utz B, Biswas A, Rahman F, van den Broek N. Cause of and contributing factors to maternal deaths; a cross-sectional study using verbal autopsy in four districts in Bangladesh. BJOG. 2014;121(Suppl 4):86–94.  Mahato PK, Waithaka E, van Teijlingen E, Raj Pant P, Biswas A. Social autopsy: a potential health-promotion tool for preventing maternal mortality in low-income countries. WHO South-East Asia J Public Heal. 2018;7(1):123–95.  Waiswa P.; Kalter H.D.; Jakob R.; Black R.E. (2012). Increased use of social autopsy is needed to improve maternal, neonatal and child health programmes in low-income countries. Bulletin of the World Health Organization, 90. |

*Source: Articles identified by Kinney et al. 2021^1^ and through the MPDSR Global Technical Working Group.*

## Panel S1. Example of principles of facility-based case review meetings to ensure no blame

Key principles:

- Meetings should be multidisciplinary and interactive. They should not be didactic. This is best achieved with participants sitting in a circle.
- Meetings should be held on a regular basis and during protected time reserved for staff attendance.
- Administrators and others who can act on the recommendations should be present.

Example meeting agenda:

1. Read and agree code of conduct.
2. Re-evaluate the recommendations of previous sessions and provide a short follow-up of actions decided at the last meeting. Identify any further action required.
3. Present the clinical summary of case(s).
4. Conduct a systematic case review, using a common template and with reference to any clinical guidelines or standards available.
5. Prepare a case analysis and local recommendations.
6. Plan for implementation.
7. Prepare a case analysis and a report to be sent to the overall steering committee for the wider review programme, if one exists.
8. Provide feedback on general findings and recommendations to staff who could not attend and to hospital administrators.

*Source: Lewis G. Emerging lessons from the FIGO LOGIC initiative on maternal death and near-miss reviews. Int J Gynaecol Obstet. 2014;127(Suppl 1):S17–20.*

## Panel S2. Examples of audit charter or non-disclosure agreements

### Generic example

Non-disclosure confidenitality agreement

We, the members of the ---- review committee, agree to maintain anonymity and confidentiality for all the cases discussed at this meeting, held on [DATE]. We pledge not to talk to anyone outside this meeting about details of the events analysed here, and will not disclose the names of any individuals involved, including family members or health-care providers.

*Source: E4A. 2012. Maternal death surveillance and response systems: overcoming legal challenges and creating an enabling environment. MDSR Action*

*Network. Presented during ‘Interactive MDSR Resource Room’ at XXFIGO World Congress of Gynecology and Obstetrics, Rome, Italy, 7-12 October 2012.*

1. Example from Burkina Faso

**Audit charter for the maternity unit of the district hospital *Secteur 30***

The medical audit consists in a systematic and critical analysis of the quality of care by comparison to defined standards (norms and care protocols). It enables the members of a team to discuss and question or improve certain practices. The audit must never be used to sanction a member of staff. Its purpose is to propose recommendations and actions aimed at avoiding in future the deficiencies or errors observed.

We, staff of the maternity of the hospital *Secteur 30*, promise to respect the rules of good practice that follow:

1. To arrive on time for audit sessions.
2. To respect the statements and ideas of everyone.
3. To respect the confidentiality of the team discussions. Information and problems raised during the audit must not be communicated outside the team (friends, relatives, colleagues in other health departments, etc.).
4. To participate actively in the discussions.
5. To accept discussion and debate among participants without verbal violence.
6. To refrain from hiding or falsifying information that could be useful in understanding the case being audited.
7. To try as much as possible (because it is not easy) to accept questioning of one’s own actions.

Staff of the maternity department, Ouagadougou, 25 February 2004

*Source: Richard F, Oue´draogo C, Zongo V, Ouattara F, Zongo S, Grue´nais M, De Brouwere V. The difficulty of questioning clinical practice: experience of facility-based case reviews in Ouagadougou, Burkina Faso. BJOG. 2009;116:38–44.*

## Panel S3: Engaging the community to prevent blame

Community awareness, sensitization and engagement around the MPDSR process can reduce blame and improve implementation. The blame culture at the community level can work in many ways. Health care workers may be blamed by women, their families and the community after a death; likewise women, their families or even communities may be blamed for deaths related to delaying care.^2 3^ Promoting a collaborative partnership approach to the MPDSR process can be established by facilitating dialogue between community members, health care providers and managers to build trust and form learning communities. Successful community consultation and engagement on the response portion of MPDSR has shown to improve implementation of actions in some settings.^4-7^ Breaking down barriers associated with blame is especially important to strengthen community engagement around data collection of deaths in the community.^8-10^ In addition, those implementing MPDSR need to acknowledge and accommodate for the strong emotions associated with death, such as grief and anger.  As such, the actors (both community and health care providers) need to be empathetic in validating and carefully managing these emotions by providing support to the bereaved families, such as psychosocial support relevant to the context.

REFERENCES

1. Kinney MV, Walugembe DR, Wanduru P, Waiswa P, George A. Maternal and perinatal death surveillance and response in low- and middle-income countries: a scoping review of implementation factors. *Health policy and planning* 2021 doi: 10.1093/heapol/czab011.

2. E4A. Maternal death surveillance and response systems: overcoming legal challenges and creating an enabling environment: MDSR Action Network 2012. Available from: <http://mdsr-action.net/wp-content/uploads/2015/08/E4A_2012_FIGO-legal-briefing.pdf>.

3. Melberg A, Mirkuzie AH, Sisay TA, Sisay MM, Moland KM. 'Maternal deaths should simply be 0': politicization of maternal death reporting and review processes in Ethiopia. *Health policy and planning* 2019;34(7):492-98. doi: 10.1093/heapol/czz075.

4. Abebe B, Busza J, Hadush A, Usmael A, Zeleke AB, Sita S, et al. 'We identify, discuss, act and promise to prevent similar deaths': a qualitative study of Ethiopia's Maternal Death Surveillance and Response system. *BMJ global health* 2017;2(2):e000199. doi: 10.1136/bmjgh-2016-000199.

5. Hofman JJ, Mohammed H. Experiences with facility-based maternal death reviews in northern Nigeria. *Int J Gynaecol Obstet* 2014;126(2):111-4. doi: 10.1016/j.ijgo.2014.02.014.

6. Kerber KJ, Mathai M, Lewis G, Flenady V, Erwich JJ, Segun T, et al. Counting every stillbirth and neonatal death through mortality audit to improve quality of care for every pregnant woman and her baby. *BMC Pregnancy Childbirth* 2015;15 Suppl 2:S9. doi: 10.1186/1471-2393-15-S2-S9.

7. Kongnyuy EJ, Leigh B, van den Broek N. Effect of audit and feedback on the availability, utilisation and quality of emergency obstetric care in three districts in Malawi. *Women and birth : journal of the Australian College of Midwives* 2008;21(4):149-55. doi: 10.1016/j.wombi.2008.08.002.

8. Kerber KJ, Mathai M, Lewis G, Flenady F, Erwich JJHM, Segun T, et al. Counting every stillbirth and neonatal death to improve quality of care for every pregnant woman and her baby. *BMC Preg Childbirth* 2015;15(Suppl 2)(S9).

9. Agaro C, Beyeza-Kashesya J, Waiswa P, Sekandi JN, Tusiime S, Anguzu R, et al. The conduct of maternal and perinatal death reviews in Oyam District, Uganda: a descriptive cross-sectional study. *BMC Womens Health* 2016;16:38. doi: 10.1186/s12905-016-0315-5.

10. Mogobe KD, Tshiamo W, Bowelo M. Monitoring maternity mortality in Botswana. *Reprod Health Matters* 2007;15(30):163-71. doi: 10.1016/S0968-8080(07)30330-3.
